# High-Temperature Hydrothermal Extraction of Phenolic Compounds from Brewer’s Spent Grain and Malt Dust Biomass Using Natural Deep Eutectic Solvents

**DOI:** 10.3390/molecules29091983

**Published:** 2024-04-25

**Authors:** Dries Bleus, Heike Blockx, Emma Gesquiere, Peter Adriaensens, Pieter Samyn, Wouter Marchal, Dries Vandamme

**Affiliations:** 1 Analytical and Circular Chemistry (ACC), Institute for Materials Research (IMO-IMOMEC), Hasselt University, Agoralaan, 3590 Diepenbeek, Belgium; 2 Department of Circular Economy and Renewable Materials, Sirris, Gaston Geenslaan 8, 3001 Leuven, Belgium

**Keywords:** natural deep eutectic solvent, green solvent, biomass valorisation, hydrothermal extraction, phenolic compounds

## Abstract

Aligned with the EU Sustainable Development Goals 2030 (EU SDG2030), extensive research is dedicated to enhancing the sustainable use of biomass waste for the extraction of pharmaceutical and nutritional compounds, such as (poly-)phenolic compounds (PC). This study proposes an innovative one-step hydrothermal extraction (HTE) at a high temperature (120 °C), utilizing environmentally friendly acidic natural deep eutectic solvents (NADESs) to replace conventional harmful pre-treatment chemicals and organic solvents. Brewer’s spent grain (BSG) and novel malt dust (MD) biomass sources, both obtained from beer production, were characterized and studied for their potential as PC sources. HTE, paired with mild acidic malic acid/choline chloride (MA) NADES, was compared against conventional (heated and stirred maceration) and modern (microwave-assisted extraction; MAE) state-of-the-art extraction methods. The quantification of key PC in BSG and MD using liquid chromatography (HPLC) indicated that the combination of elevated temperatures and acidic NADES could provide significant improvements in PC extraction yields ranging from 251% (MD-MAC-MA: 29.3 µg/g; MD-HTE-MA: 103 µg/g) to 381% (BSG-MAC-MA: 78 µg/g; BSG-HTE-MA: 375 µg/g). The superior extraction capacity of MA NADES over non-acidic NADES (glycerol/choline chloride) and a traditional organic solvent mixture (acetone/H_2_O) could be attributed to in situ acid-catalysed pre-treatment facilitating the release of bound PC from lignin–hemicellulose structures. Qualitative ^13^C-NMR and pyro-GC-MS analysis was used to verify lignin–hemicellulose breakdown during extraction and the impact of high-temperature MA NADES extraction on the lignin–hemicellulose structure. This in situ acid NADES-catalysed high-temperature pre-treatment during PC extraction offers a potential green pre-treatment for use in cascade valorisation strategies (e.g., lignin valorisation), enabling more intensive usage of available biomass waste stream resources.

## 1. Introduction

Driven by Green Chemistry principles [1] and EU Sustainable Development Goals 2030 [2], considerable research efforts are focused on creating circular processes to replace traditional ‘linear’ industrial production methods in an attempt to intensify industry: more production with less energy, less resources, and less waste [3,4]. A major contributor to this transition is the valorisation of waste materials and side streams, particularly biomass side streams from the agricultural and food industries. These side streams can be seen as a complex biochemical matrix containing countless valuable nutrients and bio-active compounds, potential bio-precursors for synthesis, etc. [5]. A class of extractable compounds that are currently under extensive research are (poly-)phenolic compounds (PC) [6]. The antioxidant properties of these biomolecules offer promising potential health benefits as food additives [7] and even for the treatment and prevention of cancer and other diseases [8,9]. Therefore, developing and improving methods for the (partial) valorisation of these biochemical compounds from biomass is a pivotal field of research. To adhere to new governance rules, current extraction methods with traditional and often harmful organic solvents and harsh chemical pre-treatments should be phased out in favour of novel, more efficient methods utilising green solvents [10].

Malt dust (MD), a novel and underutilized biomass side stream, is investigated in this study. Comprising about 1% of total malt produced, MD is a fine powder residue which is separated from the malted and dried barley after the malting stage [11]. It, therefore, remains unused in subsequent beer production steps, retaining potential nutritional compounds. MD presents an untapped biomass stream for further valorisation. MD has been effectively utilized in enzymatic lactic acid production for beer acidification [11] and demonstrated promise for conversion to monosaccharides and organic acids through acid- and base-catalysed hydrolysis [12]. However, to the best of the authors’ knowledge, the usage of MD for the green extraction of PC is a novel valorisation pathway that has not yet been explored in the literature. The extraction of PC from MD is compared to another, more established biomass side stream from beer production: brewer’s spent grain (BSG) [13,14,15].

A generally observable trend in current state-of-the-art biomass extraction methods is the overall increase in extraction efficiency when incorporating a pre-treatment step using traditional harsh acids or bases such as H_2_SO_4_ and NaOH [16,17]. Secondly, similar improvements can be observed at higher extraction temperatures, showing marked increases in extraction yields at temperatures closer to or at 100 °C [17,18]. However, most experimental methodologies found in the literature are typically performed at temperatures ≤100 °C. Higher temperatures are stipulated to provide better solvent mobility, and pre-treatments create a certain degree of lignocellulosic matrix breakdown, both offering favourable effects for the extraction of PC that are retained within the lignocellulosic structure (‘bound’ PC represent the majority of the total estimated phenolic composition) [16]. For the first time, to the best of the authors’ knowledge, a novel extraction method, combining elevated temperatures through the use of a hydrothermal reactor with green, natural deep eutectic solvents (NADES), is proposed in this research.

Deep eutectic solvents (DES) are a broad class of solvent mixtures made up of Lewis or Brønsted acids and bases able to form eutectic mixtures with a freezing point far below those of the pure components [19]. NADES can be considered a sub-category of DES, specifically composed of natural plant metabolites (classified as “biosynthetically primordial metabolites, PRIM”) [20]. These plant metabolites are generally classified as non-toxic and biodegradable, all the while being inherently abundant in nature.

This offers health and safety benefits over harsher traditional non-halogenated (e.g., hexane, methanol) and halogenated (e.g., dichloromethane) organic solvents [21], many of which carry substantial toxicity or carcinogenicity risks, are highly flammable, and typically have high vapour pressures. Hence, NADES have great potential for use in extracting compounds destined for food or medicinal use. With a wide variety of NADES to choose from, the selection of a suitable composition relies on a combination of physicochemical properties, such as viscosity, acidity (of the pure components), and polarity [22,23].

NADES viscosity, and correlated solvent mobility, is an important parameter to be optimised, as NADES are typically characterised with viscosities several orders of magnitude higher than water or organic solvents. To improve solvent mobility, tertiary NADES mixtures are created through the addition of water. However, care must be taken to balance lowering viscosity and weakening the original H-bonding interactions of the pure NADES compounds. A general empirical consensus exists that beyond 50% (*v*/*v*), hydrogen interactions between NADES components and water outweigh the original NADES interactions [23,24]. This study intentionally stays well below this limit (10–25 *v*/*v*%) to align with green principles, minimizing water consumption compared to state-of-the-art extraction solvent mixtures that often exceed 30 *v*/*v*% H_2_O (Table 1).

Previous studies have explored the use of ‘designer’ solvents for phenolic extraction from brewer’s spent grain (BSG). However, diverse extraction methods, including maceration with heating and agitation (stirring) (MAC), microwave-assisted extraction (MAE), and ultrasound-assisted extraction (UAE), result in varying optimization paths and extraction outcomes. Table 1 provides a brief overview of commonly used methodologies for extracting phenolic compounds (PC) from BSG using both traditional and deep eutectic solvents. Extraction efficiencies are evaluated based on yields of four representative phenolic compounds: caffeic acid (CAFF), syringaldehyde (SYR), p-coumaric acid (COUM), and ferulic acid (FER).

This study explores the usage of a novel hydrothermal extraction method (HTE) at an elevated temperature of 120 °C using tertiary NADES solvents applied to brewer’s spent grain (BSG) and novel malt dust (MD) biomass. For the first time, to the best of the author’s knowledge, mild acidic NADES is used in conjunction with high-temperature extraction to increase PC extraction efficiency. For this purpose, malic acid/choline chloride (MA, 1:1 molar ratio) is selected for its balanced, mildly acidic (pKa_1_ = 3.40) properties (compared to other often-used organic weak acids for NADES: levulinic acid, pKa = 4.64; lactic acid, pKa_1_ = 3.86; citric acid pKa_1_ = 3.13; oxalic acid, pKa_1_ = 1.27) and benchmarked against glycerol/choline chloride (GLY, 2:1 molar ratio) for its well-documented efficiency at PC extraction [18,33]. The unique combination of high-temperature extraction and acidic MA NADES offers an eco-friendly alternative to harsh pre-treatments with traditional acids and bases. This green strategy paves the way for the cascade refining of biomass, envisioning further valorisation of components like cellulose or lignin [34,35]. The influence of this unique combination of extraction conditions is benchmarked against two known state-of-the-art methods. On the one hand, heated and stirred maceration (MAC) offers a similar batch extraction environment to HTE at temperatures below 100 °C, thus lacking the autogenous solvent pressures observed in HTE. Secondly microwave-assisted extraction (MAE) offers a modern approach often used for this type of PC extraction and demonstrated before for BSG [18]. Finally, the impact of the extraction method on biomass structure is investigated through ^13^C NMR and pyrolysis GC-MS to further understand how the proposed novel extraction methodology can impact PC yields and biomass valorisation.

## 2. Results and Discussion

### 2.1. Biomass Characterisation 

The BSG and MD biomass side streams used in this work were first characterized to gain some insight into their composition. Malt dust has received little attention in current research; hence, its chemical composition has not yet been studied in great detail in the past. Notable for MD, the percentage of water- and ethanol-extractable compounds represented over 58 wt% of the composition. This can be ascribed to the sugars, starches, and enzymes present in the biomass after malting, which are easily removed during the hot-water extraction. In contrast, most of the starch and sugars are removed from the malt during the mashing step; hence, BSG only had around 23 wt% of extractives.

NDF and ADF biomass characterisation according to the method of van Soest [36] was performed, followed by additional Klason lignin determination to provide context for the underestimation of lignin (acid detergent lignin; ADL) by the van Soest method [37]. ADL content of the BSG biomass was only 8.09 wt% (Table 2); the characterisation of the MD biomass was not possible using the van Soest method due to the fine particle size. The quantification of lignin using the Klason method yielded a total lignin content of 14.77 wt% for BSG, as this method accounts for acid-soluble lignin as well (6.00 wt%). MD had a much lower Klason lignin content of only 7.68 wt% (of which 3.40 wt% acid-soluble lignin) due to its much higher total extractive content compared to BSG (Table 2). When both biomasses were compared on an extractive-free basis, the Klason lignin content was much more similar: 19.23 and 18.33 wt% for BSG and MD, respectively. Variation in biomass lignin content [38,39] can also be the basis of the differences in phenolic content reported, as a large majority of (poly-)phenolics (especially ferulic acid) are strongly bound within the lignin structure [32,40].

The elemental composition of the BSG and MD streams was shown to be mostly similar, as would be expected, since both originate from the same biomass. The carbon content of BSG was about 5% higher, and the 0.49% higher nitrogen content translated to a slightly higher protein content of BSG compared to MD (25.66 and 22.93 wt%, respectively). The mineral content (ash) of BSG was found to be substantially lower than that of MD (approx. 3%) due to the leaching out of soluble minerals from the malt during the brewing process [41]. Secondly, the smaller particle size of MD could also have an inverse effect on the observed ash content of some biomass, as previously reported in reference [42].

### 2.2. NADES Viscosity Optimisation

**Figure 1:**
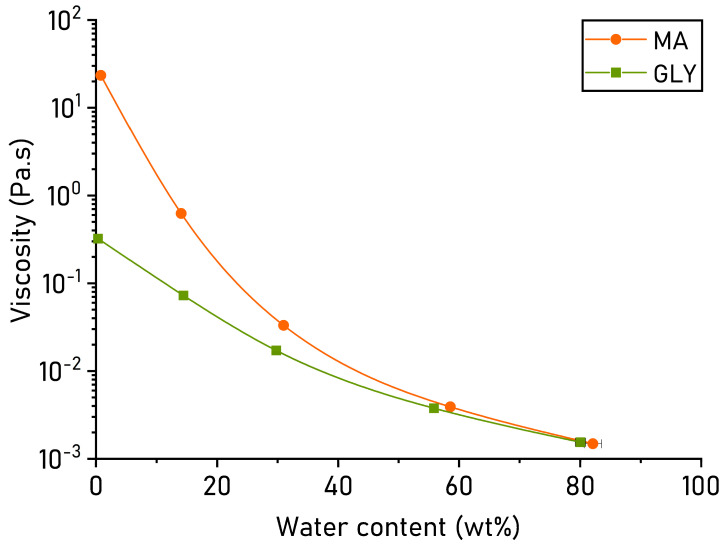
Dynamic viscosity (Pa·s) of MA and GLY, expressed in function of water content (all values reported as $\bar{x}$ ± standard error (SE); *n* = 2).

Water-free NADES suffer from extremely high viscosities, which is unfavourable for extractions where optimal solvent mobility is required. Hence, the effect of H_2_O addition on NADES viscosity was examined through rheological analysis of samples with varying water content between 0 and 75 wt% to confirm the optimal water content for the extraction solvents. Rheology measurements (dynamic viscosity) at 25 °C showed a substantially higher viscosity of water-free MA compared to water-free GLY (Figure 1). For both, dilution with 10 wt% H_2_O offers a strong reduction in viscosity. For MA, a decrease of almost two orders of magnitude is obtained, resulting in a viscosity well under 1 Pa·s. The addition of 25 wt% H_2_O roughly equalized viscosity for both MA and GLY, as it can be expected that water becomes a more dominant contributor to the hydrogen-bonding interactions. At 50 to 75 wt%, viscosities of the NADES mixtures leaned closely toward that of pure H_2_O (10^−3^ Pa·s) [43]. This confirms the predominantly water-based nature of those mixtures [23]. In conclusion, the rheological data confirm the choice of diluting both NADES solvents to a final water content of 25 wt% H_2_O as a promising composition for biomass extraction purposes. Especially at elevated temperatures, where the viscosity lowers further, it could be expected that the tertiary mixtures with 25 wt% H_2_O offer suitable solvent mobility [44].

### 2.3. Time and Temperature Stability of Phenolic Compounds 

The proposed extraction method in this work utilised markedly higher temperatures compared to conventional methods. Due to concerns about PC degradation, the effect of increased extraction temperatures and stability over time of the phenolic extract was investigated using model systems.

**Figure 2:**
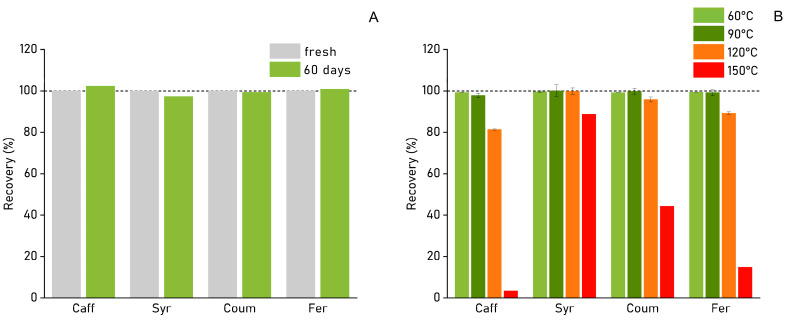
(**A**) Phenolic compound stability in solution (GLY), expressed as percentage recovery (%) relative to a reference stock solution. Recovery was measured after storing the stock solution for 60 days at 8 °C (all values reported as x; *n* = 1). (**B**) Phenolic compound degradation in GLY solution as a function of temperature, expressed as percentage recovery (%) relative to an untreated reference. Samples were heated for exactly 60 min at, respectively, 60 °C, 90 °C, 120 °C, and 150 °C (all values reported as $\bar{x}$ ± standard error (SE); *n* = 2, except for ‘150 °C’ where *n* = 1).

For time-dependent stability, a stock solution of four PCs in GLY NADES, acidified with 1% FA, was quantitatively analysed using HPLC over a 60-day time period (Figure 2A). The stability of the PC stock solutions was found to be very adequate when stored properly over time at a controlled temperature, as degradation of the four standards remained well below 3% after 60 days of storage at 8 °C. Most notably, the stability of the three HCA phenolic compounds (CAFF, COUM, and FER) was highest (>99%). With a recovery of >97%, SYR showed the highest degradation in function of time.

The effect of extraction temperatures exceeding 100 °C on phenolic stability was investigated using a 500 ppm stock solution of four indicative PCs in GLY (Figure 2B). It was found that 120 °C, the temperature conditions used in HTE, had no adverse effect on the stability of SYR and COUM (recoveries of the standards were 95% or higher). Degradation effects started to become visible at 120 °C for CAFF and FER, for which recoveries were 81.3% and 89.2%, respectively. The slight PC degradation at 120 °C was deemed an acceptable trade-off in favour of higher extraction temperatures. Strong phenolic degradation was observed at 150 °C, though notably at 150 °C, CAFF, COUM, and FER (all hydroxycinnamic acids) were more severely affected compared to syringaldehyde.

### 2.4. Phenolic Compound Extractions

**Figure 3:**
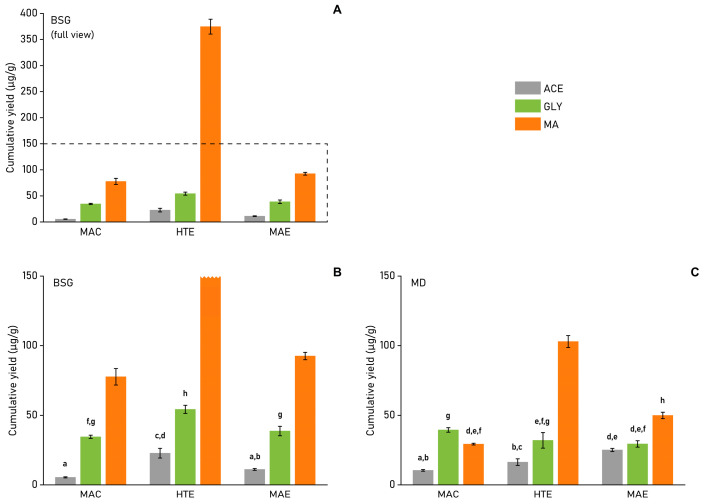
Cumulative phenolic extraction yield (sum of the four quantified main phenolic compounds; µg/g). (**A**) BSG (full view, dashed line represents *y*-axis scaling of figure (**B**)), (**B**) BSG (scaled 150 µg/g on the *y*-axis), (**C**) MD. (All values reported are on dry-weight basis, $\bar{x}$ ± standard error (SE); *n* = 2. Columns with different superscript letters, ^a–h^_,_ differ significantly (*p* < 0.05).

Firstly, looking at the ‘benchmark’ methods MAC and MAE, cumulative phenolic yields (a summation of the four identified and quantified major PC in the extracts, based on quantitative HPLC analysis) (Figure 3) indicate that the maceration method performed worse in all but MD-MAC-GLY extraction, where MAC shows remarkably high extraction yield, even compared to the hydrothermally extracted MD-HTE-GLY.

The MAE method performed favourably overall when compared to MAC extractions. Despite short extraction times being successfully reported in references [17,18], the method utilized here could not provide extraction yields similar to the best results obtained from the HTE method. It is clear that further method optimisation would be necessary to be able to reproduce state-of-the-art results, as found in references [18,28].

Apart from the single deviating result of MD-MAC-GLY, the HTE method showed improved overall extraction yields ranging from 56% (MD-MAC-ACE: 10.5 µg/g; MD-HTE-ACE: 16.4 µg/g) to 381% improvement (BSG-MAC-MA: 78 µg/g; BSG-HTE-MA: 375 µg/g). It is clear that these improvements could be attributed to the acidic extraction conditions provided by the high concentration of malic acid in the solvent mixture. These extraction conditions provide an in situ acid-catalysed MA pre-treatment, inducing (partial) destruction of the sample’s lignocellulosic matrix at 120 °C. This finding aligns with several studies that highlight the excellent applicability of carboxylic acid-based NADES in the fractionation of lignin and lignocellulosic biomass [45,46,47,48,49].

As GLY lacks the acidic character of MA, its more benign character might mean that the biomass structural matrix is mostly left intact even at 120 °C, making it less likely to extract additional phenolics typically retained within the structural matrix. Therefore, the observation of a negative trend for MD when raising the temperature from 90 °C (MAC) to 120 °C (HTE) is to be expected as phenol degradation becomes more prevalent.

While HTE conditions and the effects of acidic MA did show benefits for the extraction of MD, the overall yields indicated less strong variation between the different extraction methods and conditions compared to BSG. The observed ‘indifferent’ extraction behaviour could be due to the much smaller particle size of MD biomass. Research on cocoa bean shells [50] and grapevine residue [51] has shown that biomass particle size can heavily impact extraction efficiency. In this case, the extremely fine particles make MD easier to extract, even for less efficient extraction methods.

**Figure 4:**
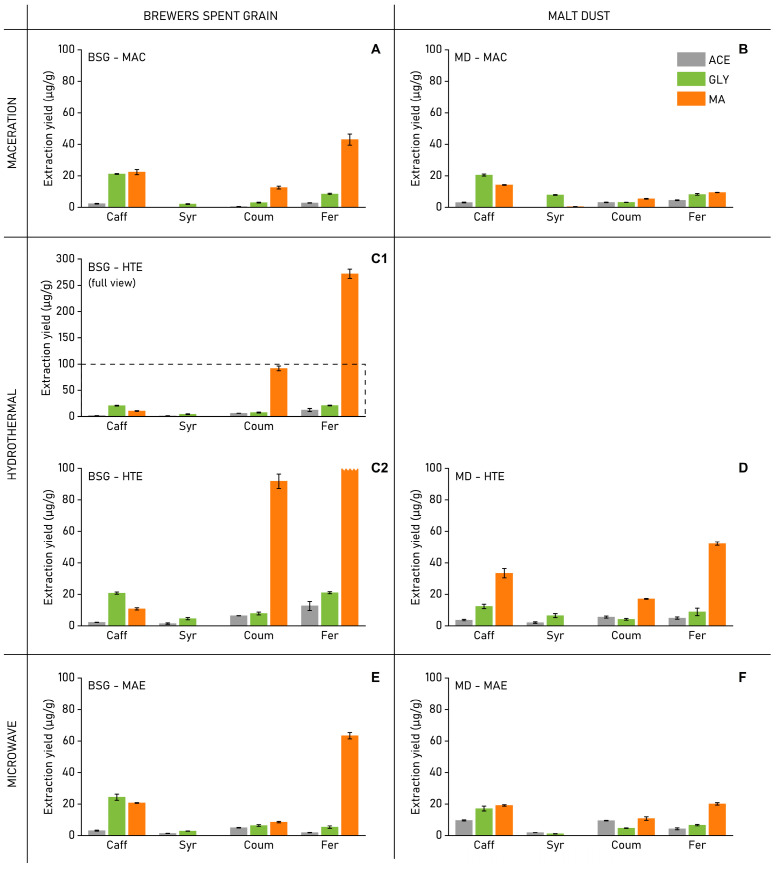
Extraction yields of the four individual phenolic compounds (i.e., CAFF, SYR, COUM, FER), quantified using HPLC data and external calibration curves for all four phenolic compounds. (**A**) BSG-MAC, (**B**) MD-MAC, (**C1**) BSG-HTE (full view, dashed line represents *y*-axis scaling of figure (**C2**)), (**C2**) MD-HTE (*y*-axis scaled to 100 µg/g), (**D**) MD-HTE, (**E**) BSG-MAE, (**F**) MD-MAE (all values reported are on dry-weight basis, $\bar{x}$ ± standard error (SE); n = 2).

Analysing the extraction yields of the four identified PCs (caffeic acid, syringaldehyde, *p*-coumaric acid, and ferulic acid) individually provides better insight into how the different extraction conditions compare (Figure 4). Firstly, MAC extractions using NADES solvents offered equal, but in most cases considerably higher, yields for all PC compared to the reference ACE solvent. Most notably, GLY was able to extract up to 21.2 µg/g caffeic acid from BSG and 20.5 µg/g for MD. This represents a 6- to 10-fold increase over ACE (BSG: 2.3; MD: 3.0 µg/g). MA was able to extract 43 µg/g of ferulic acid from BSG, compared to 8.5 µg/g for GLY and only 2.7 µg/g for ACE.

Malic acid-based NADES were most effective in extracting hydroxycinnamic acid PC (CAFF, COUM, and FER). Using this solvent, the most significant yield improvements are observable for COUM and FER, which represent the majority of PC found in BSG [16]. As these two phenolic acids are typically bound to lignin and hemicellulose structures [52], the MA-catalysed lignocellulosic breakdown offered an effective method to free up and extract these compounds. For brewer’s spent grain, the HTE method with MA offered a yield of 92 µg/g COUM and 272 µg/g FER, compared to 12.5 µg/g and 43 µg/g, respectively, when using the same solvent in the MAC extraction. In the case of malt dust, HTE with MA could extract 17.2 µg/g COUM and 52 µg/g FER, compared to, respectively, 5.3 µg/g and 9.41 µg/g using MAC. Microwave-assisted extractions using MA offered improved FER yields (BSG-MAE-MA: 63 µg/g; MD-MAE-MA: 20.1 µg/g) compared to extractions combining the MAC method with MA but remained substantially less efficient compared to the HTE method.

### 2.5. Impact of Acidic NADES Extraction Using HTE on the Lignocellulosic Composition of Biomass 

The high extraction yields of BSG-HTE-MA (and, to a lesser extent, MD-HTE-MA) warranted further compositional analysis of the extracted BSG to confirm the finding of acid-catalysed breakdown of the lignocellulosic matrix.

When comparing py-GC-MS data (See Supplementary Material S1) of raw BSG, BSG-HTE-GLY, and BSG-HTE-MA, changes in the lignocellulosic composition of BSG can be observed. A reduced presence of markers for hemicellulose breakdown fragments (e.g., acetic acid, furfural, 1-hydroxy-2-propanone) was most apparent alongside drastically lowered concentrations of lignin-based phenolic breakdown fragments (e.g., creosol, 4-ethyl-2-methoxy phenol, 4-ethenyl-2,6-dimethoxy-phenol). Both of which indicate structural changes occurring in the hemicellulose and lignin fractions [53].

**Figure 5:**
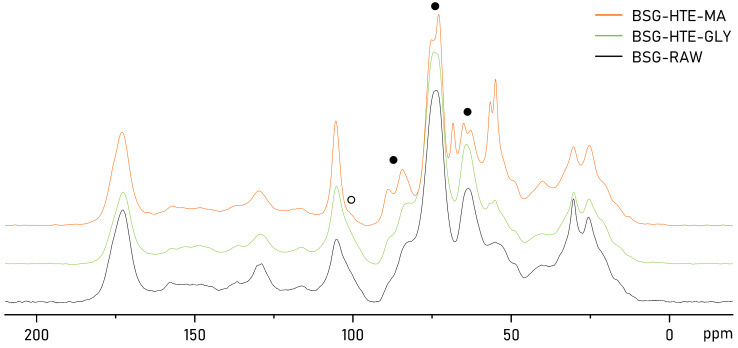
^13^C NMR of BSG before extraction (BSG-RAW), compared to BSG after extraction using HTE (BSG-HTE-GLY, BSG-HTE-MA).

Looking at the solid-state ^13^C NMR spectra of raw BSG, BSG-HTE-GLY, and BSG-HTE-MA (Figure 5), similar conclusions could be drawn. Solid-state NMR is a powerful technique to study polymers and polymer networks [54]. While changes in lignin content were difficult to interpret from the less-defined and cluttered aromatic region (roughly 110–165 ppm), more distinct changes can be observed in the 55–110 ppm region, where signals for the C_1–6_ carbon atoms of (hemi-)cellulose are located [55]. These signals are typically quite broad for biomass, as the broader hemicellulose and sharper cellulose peaks strongly overlap (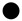). However, more defined, sharper peaks were observed in the spectrum of BSG-HTE-MA. This can only be explained by the removal of hemicellulose during the extraction, leaving only the sharper peaks from cellulose [56].

Supporting this conclusion is the (almost complete) disappearance of the broad shoulder at 100 ppm in the spectrum of BSG-HTE-MA (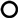). This signal corresponds to the C1 signal of hemicellulose and overlaps partially with the C1 signal from cellulose around 105 ppm [57].

## 3. Materials and Methods

### 3.1. Chemicals and Reagents 

BSG or MD biomass were obtained from Alken-Maes brewery (Alken, Belgium) and immediately dried at 65 °C until the residual water content was approximately at or below 5 wt%. Malic acid, glycerol, and choline chloride, used to prepare NADES formulations, were obtained from Thermo Fischer Scientific (Waltham, MA, USA). For the HPLC analysis, ferulic acid (FER; Thermo Scientific, Waltham, MA, USA), caffeic acid (CAFF; Thermo Scientific, Waltham, MA, USA), *p*-coumaric acid (COUM; Thermo Scientific, Waltham, MA, USA), and syringaldehyde (SYR; TCI chemicals, Tokyo, Japan) were used as standards for prevalent phenolic compounds. LC-grade formic acid (LiChropur; Merck, Darmstadt, Germany) was used to acidify the samples for HPLC analysis. Analytical reagent grade acetone (AnalaR NORMAPUR) and analytical reagent grade methanol (HiPerSolv CHROMANORM for LC-MS) were obtained from VWR (Radnor, PA, USA). Ultrapure (UP) water (18 MΩ·cm) was obtained using an Arium Pro System (Sartorius, Göttingen, Germany). Fresh UP water was prepared every day to prevent impurities.

### 3.2. Biomass Characterisation 

#### 3.2.1. Component Analysis 

The untreated biomass samples were analysed for their composition based on the acid detergent fibre (ADF) and neutral detergent fibre (NDF) method adapted from Van Soest and Wine [36], with an additional ethanol pre-processing step (according to ASTM E 1690–01) to remove ethanol-extractable components (include waxes, fats, resins, tannins, gums, sugars, starches, and pigments) [58]. Van Soest analysis for the MD biomass was not possible, as the particle size of this biomass was too small to be retained on sintered glass filters, required for the oxidative treatment step in the Van Soest and Wine method.

Additionally, for both BSG and MD samples, Klason lignin quantification was performed based on a standardized protocol by NREL (NREL/TP-510-42618) preceded by a two-stage Soxhlet extraction to remove extractives (NREL/TP-510-42619).

#### 3.2.2. Ultimate Analysis 

Elemental composition (carbon, hydrogen, nitrogen, and sulphur contents) of both BSG and MD was determined using a Thermo Electron Flash EA1112 elemental analyser (Thermo Fischer Scientific, Waltham, MA, USA). The instrument was calibrated with a reference standard: BBOT ((2,5-bis (5-tert-butyl-benzoxazole-2-yl) thiophene) (Thermo Fischer Scientific, Waltham, MA, USA). For each analysis, between 3 and 4 mg of sample was weighed. All samples were measured in quadruplicate. Protein content was derived indirectly from the nitrogen content present in the sample, based on a standardized protocol by NREL (NREL/TP-510-42625) using a biomass-specific conversion factor (nitrogen factor, NF). A reference NF value of 5.49 is used in this study based on the literature values for barley [59].

#### 3.2.3. Gravimetric Ash Content 

According to ASTM D2866–94 standard protocols [60], ash content of BSG and MD was determined gravimetrically by ashing ±1 g of dry biomass at 650 °C for 3 h in a muffle furnace. Total oxygen content could then be determined indirectly according to the following equation:

(1) $$O\left(\mathrm{wt}\%\right)=100\%-C\left(\mathrm{wt}\%\right)-N\left(\mathrm{wt}\%\right)-H\left(\mathrm{wt}\%\right)-S\left(\mathrm{wt}\%\right)-\mathrm{Ash}\left(\mathrm{wt}\%\right)$$

Sulphur contents of the biomass samples were below the detection limits (<0.5 wt%).

### 3.3. NADES Preparation 

Two different tertiary NADES were prepared using a heating method. Malic acid/choline chloride (MA) NADES with a 1:1 molar ratio and glycerol/choline chloride (GLY) NADES with a 2:1 molar ratio. For both NADES compositions, the pure constituents were combined and diluted with water to a final water content of 25 wt%. The mixtures were then stirred at 50 °C for 1 h until fully dissolved and clear. NADES mixtures were finally agitated in an ultrasonic bath at 50 °C for 30′ to finalize dissolution and degassing. An acetone/H_2_O solvent mixture (ACE) was prepared in a 60:40 volume/volume ratio as a classical benchmark extraction solvent [25].

### 3.4. Time- and Temperature-Dependent Phenolic Compound Stability Test 

For the time stability test, a stock solution of 500 ppm of CAFF (500 mg/L), COUM acid (500 mg/L), FER (500 mg/L), and SYR (500 mg/L) was made in GLY NADES and stored at 8 °C immediately after preparation for 60 days. Stability was assessed through HPLC analysis. Concentrations of the phenolic compounds were quantified based on the integrated peak area using an external calibration curve of the four phenolic compounds.

To assess the temperature stability of the phenolic compounds, 1 mL aliquots of this 500 ppm phenolic stock solution were made in duplicate in closed HPLC vials. Samples were heated for 1 h at 60 °C, 90 °C, 120 °C, and 150 °C in a heating block, followed by cooling down and storage at 8 °C until quantitative analysis of the phenolic content by HPLC. Concentrations of the phenolic compounds after thermal treatment were quantified based on the integrated peak area using an external calibration curve of stock solutions (0, 100, 250, 500 ppm) of the four phenolic compounds.

### 3.5. Solvent Viscosity Determination, Rheology 

Prior to rheological analysis, the water content of all NADES samples was determined in duplicate using a Karl-Fischer titration with a Titroline 7500KF automatic Karl-Fischer setup (SI Analytics, Mainz, Germany). Rheological analysis was performed on an AR-G2 air bearing rheometer (TA Instruments, New Castle, DE, USA), equipped with a TA Instruments 40 mm 2° angle stainless steel cone (511,406.901; TA Instruments, New Castle, DE, USA). Viscosity characteristics were determined based on a steady-state flow measurement using constant rotation speeds varied between 10^−2^ and 10^3^ s^−1^. Measurements were performed in duplicate. Dynamic viscosities were determined from the resulting viscosity curves at a shear rate of 100 s^−1^ (or 1 s^−1^ for sample MA 0 wt% H_2_O due to high viscosity). Data were smoothed using TA Data Analysis software (v5.7.0, TA Instruments, New Castle, DE, USA) to remove outlier datapoints in the low-shear regions, which did not influence the steady-state section of the measurements.

### 3.6. Biomass Extractions 

**Scheme 1:**
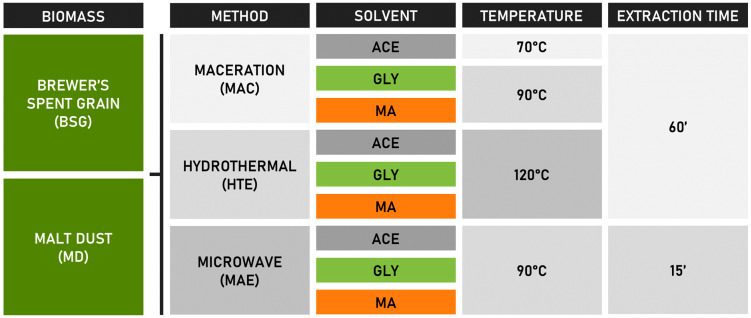
Schematic overview of biomass extractions performed in this research.

An overview of the extraction methodology is given in Scheme 1. All extractions were performed using a 1:20 (*w*/*w*) biomass (dry)-to-solvent ratio, which is a common and optimized ratio reported in various studies [16,17,25]. Extraction time and temperatures for the reference methods (MAC and MAE) were adapted from optimized PC extraction methods described in the literature [18,25]. Sample names used in the Discussion Section are based on a combination of the abbreviations for biomass + extraction method + solvent. e.g., brewer’s spent grain extracted using maceration with glycerol-based NADES:BSG-MAC-GLY.

#### 3.6.1. Heated and Agitated (Stirred) Maceration Extraction (MAC) 

The heated and stirred MAC extraction conditions were adapted from reference [25]: 3 g of biomass was combined with 60 g of NADES in 100 mL screw-cap closed vessels. The extraction was performed in a heated water bath for 60 min at 90 °C, under constant stirring at 500 rpm, and carried out in quadruplicate. As a benchmark comparison experiment, this setup was repeated with ACE as solvent, performed at 70 °C (just below the theoretic boiling point of the acetone/H_2_O mixture).

#### 3.6.2. Hydrothermal Extraction (HTE) 

A series 5500 HP 400 mL compact reactor (Parr, Moline, IL, USA) was used to obtain BSG and MD extracts. In a pressure-resistant stainless-steel Parr reactor with glass liner, 10 g of dry BSG or MD biomass was mixed with 200 g of solvent (ACE, GLY, or MA). The temperature was set at 120 °C, and the stirring motor velocity was set at 260 rpm. After reaching the target temperature, the HTE was performed for 60 min. Pressures reached 0.8 and 4.6 bar above atmospheric pressure for NADES (MA and GLY) and ACE, respectively. The reaction vessel was cooled down to ambient temperature before removing the extract. All extractions were carried out in duplicate.

#### 3.6.3. Microwave-Assisted Extraction (MAE) 

MAE of BSG and MD was performed using an Ethos up microwave digestion system (Milestone, Sorisole, Italy) equipped with glass-lined Teflon closed-cap organic solvent extraction vessels (Fastex; Milestone, Sorisole, Italy). The extraction conditions were adapted from reference [18]. In the extraction vessels, 2 g of dry biomass was combined with 40 g of solvent (ACE, GLY, or MA). The microwave program was set at a 15 min isothermal period at a temperature of 90 °C, and magnetic stirring was used to agitate the sample during extraction (stirring intensity 40%). All extractions were carried out in duplicate. As a baseline experiment, this setup was repeated with ACE as solvent.

### 3.7. Work-Up Extracts 

A small aliquot of extract was decanted into Falcon tubes and centrifuged twice at 9000 rpm for 5 min with a Centrifuge 5804R (Eppendorf, Hamburg, Germany). The supernatant was stored in 20 mL vials for storage at −18 °C until quantitative analysis of the phenolic content by HPLC.

### 3.8. High-Performance Liquid Chromatography (HPLC) 

HPLC was used to identify and quantify PC present in the BSG and MD extraction samples. The samples containing NADES were 1:1 diluted with methanol/water (MeOH:H_2_O) containing 2% formic acid (FA). To all other samples, HPLC-grade FA was added to obtain a final concentration of 1% FA. All NADES samples were filtered through PTFE syringe filters (0.45 µm). Four PC abundantly present in BSG were selected to be identified and quantified in the extracts: i.e., caffeic acid (CAFF), *p*-coumaric acid (COUM), ferulic acid (FER), and syringaldehyde (SYR) [18]. A stock solution mixture containing the four compounds was made with a concentration of 100 ppm in MeOH:H_2_O (50:50 *v*/*v*%). This stock solution was diluted (10; 1; 0.5; 0.2; and 0.1 ppm) with MeOH:H_2_O 1% FA to construct a calibration curve.

HPLC separation was performed on a U-HPLC setup containing an Ultimate 3000 RS diode array detection unit (210 nm, 254 nm, 280 nm, 320 nm, and 210–600 nm (scan); Thermo Fischer scientific Dionex, Sunnyvale, CA, USA) and coupled to a Thermo scientific LCQ fleet (Thermo Scientific, Waltham, MA, USA) LC-MS. Samples (injection volume 20 µL) were injected on a Synergi hydro-RP C18 column (150 mm × 4.6 mm, 4 µm; Phenomenex, Torrance, CA, USA). The gradient elution method was adapted from reference [17] using acidified milli-Q water (1% FA, solvent A) and acidified MeOH (1% FA, solvent B), using the following gradient: 0–10 min, 10% B; 10–95 min, 10–95% B, 95–115 min, 95% B; 115–125 min, 95–10% B; 125–135 min, 10% B. HPLC chromatograms were analysed using Xcalibur Qualbrowser software (v2.2, Thermo Fischer Scientific, Waltham, MA, USA) and phenolic content were expressed as µg per g dry biomass weight (µg/g dw). Based on the calibration curve method proposed by the International Committee of Harmonization (ICH) [61], the limit of detection (LOD) and limit of quantification (LOQ) were determined as 0.30 µg/g dw and 0.89 µg/g dw, respectively.

### 3.9. Pyrolysis GC-MS

Py-GC-MS was performed on a Multi-Shot pyrolizer EGA/PY-3030 D (Frontier lab, Fukushima, Japan) coupled to a Gas Chromatograph Trace 1300 (Thermo Fischer Scientific, Waltham, MA, USA) with a single quadrupole mass spectrometer ISQ 7000 detector (Thermo Fischer Scientific, Waltham, MA, USA). Analysis was performed on following BSG samples before and after extraction: untreated BSG (further referred to as ‘BSG-RAW’), BSG-HTE-GLY, and BSG-HTE-MA. Between 0.3 and 0.4 mg of dry, homogenous sample was injected in a flash pyrolysis oven for a two-step pyrolysis (300 °C torrefaction, followed by 550 °C pyrolysis). Sample gasses (injection volume 20 µL) were injected into an Ultra Alloy-1 column (30 m × 0.25 mm, 0.5 µ; Frontier Lab, Fukushima, Japan) using a 1:80 split ratio. The oven temperature was set at 35 °C (1 min isothermal) and increased to 320 °C (2.5 min isothermal) using a heating rate of 10 °C/min. MS parameters were scan range of 30–550 *m*/*z*, dwell time of 0.2 s, and ion source temperature at 300 °C.

### 3.10. Nuclear Magnetic Resonance (NMR) 

^13^C solid-state CP/MAS NMR spectra were acquired on an Agilent VNMRS DirectDrive 400 MHz spectrometer (Agilent Technologies, Santa Clara, CA, USA) equipped with a T3HX 3.2 mm probe. Analysis was performed on the following BSG samples before and after extraction: BSG-RAW, BSG-HTE-GLY, and BSG-HTE-MA. Magic angle spinning (MAS) was performed at 17 kHz with ceramic rotors of 3.2 mm in diameter (34 µL rotors). The aromatic signal of hexamethylbenzene was used to determine the Hartmann–Hahn condition (ω_1H_ = γ_H_ B_1H_ = γ_C_ B_1C_ = ω_1C_) for cross-polarization (CP) and to calibrate the carbon chemical shift scale (132.1 ppm). Acquisition parameters used were the following: a spectral width of 50 kHz, a 90° pulse length of 3.1 µs, a spin-lock field for CP of 80 kHz, a contact time for CP of 1 ms, an acquisition time of 15 ms, a recycle delay time of 3 s and about 80,000 accumulations. High-power proton dipolar decoupling during the acquisition time was set to 80 kHz [54].

### 3.11. Statistical Analysis

Statistical analysis of the total cumulative PC yields (determined as the sum of all four identified PCs per extraction method) was performed using SPSS software (version 29.0, Chicago, IL, USA). One-way analysis of variance (ANOVA), in combination with a post hoc Tukey’s test, was used to determine significant (*p* values < 0.05) differences between PC yields.

## 4. Conclusions

This study has demonstrated the effectiveness of using NADES in combination with a hydrothermal extraction (HTE) at 120 °C for the extraction of phenolic compounds from lignocellulosic biomass matrices. Besides brewer’s spent grain (BSG), the novel malt dust (MD) biomass could be valorised for the first time through the extraction of phenolic compounds using the proposed method; however, the phenolic content was found to be slightly lower than that of BSG. The acidic malic acid-based NADES (MA), combined with the high extraction temperature provided by the HTE method, was able to (partially) solubilize lignin and hemicellulose and release large quantities of phenolic compounds (particularly *p*-coumaric acid and ferulic acid) from the lignocellulosic matrix of the biomass, offering greatly increased extraction yields compared to other methods and solvent systems. The partial breakdown of lignin and hemicellulose could be observed through the characterisation of pre- and post-extraction BSG. As such, the described method could offer great potential for implementation in cascade valorisation strategies for biomass, enabling multi-level valorisation; e.g., as a pre-treatment stage for lignin valorisation. While significantly improved extraction yields could be obtained using the proposed method, future research could be performed to optimize the extraction capability (e.g., by performing extractions above autogenous pressures or optimizing temperature range versus PC degradation).

## Figures and Tables

**Table 1:** Overview of some commonly used extraction procedures for BSG and their respective (poly-)phenolic compound extraction yields (all experimental values are expressed, converted, if necessary, as µg/g). (Abbreviations: total phenolic content (TPC); caffeic acid (CAFF); syringaldehyde (SYR); p-coumaric acid (COUM); and ferulic acid (FER); microwave-assisted extraction (MAE); ultrasound-assisted extraction (UAE)).

Ref.	Author	Optimized Extraction Method	Tot. Phen. (µg/g) (TPC)	CAFF. (µg/g)	SYR. (µg/g)	COU. (µg/g)	FER. (µg/g)	Notes
[25]	A. Zuorro et al., 2019	Maceration; 60 °C, 60′. Stirred 400 rpm. 1:20 *w*/*v* ratio. 0, 20, 40, 60, 80, 100 *v*/*v*% aqueous acetone and aqueous ethanol.	4126	/	/	/	/	Total yield after 4 extraction cycles. Best yield at 60 *v*/*v*% acetone.
[15]	A. McCarthy et al., 2013	Maceration (Schütz, Kammerer, Carle, & Schieber, 2004); 2 g dry BSG, 60 *v*/*v*% aqueous methanol, 90′.	4530	1.33–2.78	/	18.83–42.66	62.92–142.29	
[26]	N. Meneses et al., 2013	Maceration; 1:20 *w*/*v* ratio. 60 *v*/*v*% aqueous methanol, 30′, 60 °C–80 °C.	9900	/	/	/	/	
[16]	S. Mussatto et al., 2006	H_2_SO_4_ pre-treatment; Maceration; 2% NaOH, 1:20 *w*/*v* ratio, 120 °C, 90′.	/	/	/	2776	2906	Hydrolysis of lignin releases bound (poly-)phenols. Total solubilisation of Ferulic acid.
[27]	A. Andres et al., 2020	Maceration; 1:10 *w*/*v* ratio, H_2_O, 30 °C, 121.9′.	5420	1.69 ± 1.65	/	0.27 ± 0.06	0.88 ± 0.11	
[28]	M. Moreira et al., 2013	MAE; 0.75 *w*/*v*% NaOH.	19,500	/	/	47 ± 7	149 ± 7	
[17]	M. Moreira et al., 2012	MAE; 0.75 *w*/*v*% NaOH. 1:20 *w*/*v* ratio, 100 °C, 15′.	/	/	/	/	13,100	
[18]	J. López-Linarez et al., 2020	MAE; 1:10 *w*/*v* ratio, 100 °C, 13.3′. ChCl:GLY NADES, 37.46 *v*/*v*% H_2_O	2890	/	405	595	763	
[29]	E. Zago et al., 2022	MAE pre-treatment; 600 W, 30′. Maceration Ethanol/water 70:30 *v*/*v*%, 80 °C, 60′, 1:30 *w*/*v* ratio.	13,230 ± 20	/	/	9 ± 2	15.00 ± 0.02	
[30]	R. Birsan et al., 2019	UAE; 35 kHz. 0.75 *w*/*v*% NaOH. 1:20 *w*/*v* ratio, 80 °C, 30′.	16,990	/	/	371 ± 30	739 ± 22	
[31]	P. Forssell et al., 2008	Enzymatic hydrolysis; Econase CE, Spezyme CP, Depol 740, Depol 686; 1:10 *w*/*v* ratio, 50 °C, 5 h.	/	/	/	4500	5100	Enzymatic hydrolysis is effective for release of bound (poly-)phenols.
[32]	J. Robertson et al., 2011	Enzymatic hydrolysis; Econase CE, Alcalase, Depol 740 L, Depol 686 and Econase, feruloyl esterases. 66 mg/mL; 50–60 °C.	4140	/	/	/	/	

**Table 2:** Overview of component analysis and ultimate analysis data for the two biomass streams: brewers spent grain (BSG) and malt dust (MD) (all values reported on dry basis, $\bar{x}$ ± standard error (SE); *n* = 3 for component analysis, *n* = 4 for ultimate analysis and ash determination) (N.D. means not determined and <LOD indicates the measured value was below the limit of detection of the device).

	BSG	MD
**Compositional Analysis**	**Composition (wt%)**	**Composition (wt%)**
EtOH extractable	20.09 ± 1.44	24.23 ± 0.89
Cellulose	14.28 ± 1.46	/
Hemicellulose	42.16 ± 1.75	/
ADL Lignin	8.09 ± 2.89	/
Protein	25.66 ± 1.34	22.93 ± 0.26
**Klason lignin**		
H_2_O Extractives	13.25 ± 0.61	56.27 ± 1.75
Ethanol Extractives	9.93 ± 0.94	1.82 ± 0.09
Lignin	14.77 ± 1.97	7.68 ± 0.90
Acid-insoluble	8.77 ± 1.29	4.28 ± 0.51
Acid-soluble	6.00 ± 0.68	3.40 ± 0.39
**Ultimate analysis/ ash content**	**Composition (%)**	**Composition (%)**
C	47.79 ± 0.26	42.36 ± 0.30
H	6.76 ± 0.07	6.61 ± 0.07
N	4.67 ± 0.24	4.18 ± 0.05
O	33.98 ± 0.63	40.04 ± 0.46
S	<LOD	<LOD
Ash	3.96 ± 0.06	6.80 ± 0.04

## Data Availability

Data are contained within the article and Supplementary Materials.

## Supplementary Materials

The following supporting information can be downloaded at: https://www.mdpi.com/article/10.3390/molecules29091983/s1, Table S1: GC-MS of biomass before/after extraction using HTE method. Refs. [62,63,64,65,66,67,68,69] are cited in supplementary files.
