# Supplementary material for: High-Temperature Hydrothermal Extraction of Phenolic Compounds from Brewer’s Spent Grain and Malt Dust Biomass Using Natural Deep Eutectic Solvents
Source: Molecules. 2024 Apr 25;29(9):1983. doi: 10.3390/molecules29091983 (PMC11085089; doi:10.3390/molecules29091983)
Supplement: Supplementary file 1 [file molecules-29-01983-s001.zip › molecules-2942627-supplementary.pdf]

molecules

MDPI

Supplementary material

S1. GC-MS of biomass before/after extraction using HTE method

Table S1: py-GC-MS analysis of untreated BSG before extraction (BSG-RAW), compared to BSG after extraction using HTE (BSG-HTE-GLY, BSG-HTE-MA). Identified peaks are given with their relative peak areas, as well as designations of their origin (p-Cou: p-Coumaric acid; Cell: cellulose; Hemi: hemicellulose)

Torrefaction – 300 °C

RET. TIME (min.)	COMPOUND	RAW Rel. Area (%)	HTE-GLY Rel. Area (%)	HTE-MA Rel. Area (%)	ORIGIN	REFERENCE
2,041	Carbon dioxide	7.55	8.17	6.19		
2,095	Chloromethane	0.00	0.64	14.64		
2,190	Acetaldehyde	0.17	0.27	0.00	Lignin	
2,228	Methylamine, N,N-dimethyl-	0.00	0.00	6.88	*p*-Cou	[62]
2,269	Methanethiol	0.21	0.42	0.84		
2,296	Methylamine, N,N-dimethyl-	0.00	0.97	0.00		
2,473	Butane	0.00	0.35	0.00		
3,337	Acetic acid	4.50	4.87	0.71	Cell/ Hemi	
3,823	2-Propanone,1-hydroxy-	0.30	0.37	0.00	Cell/ Hemi	[63]
3,843	2-Chloroethanol	0.00	0.00	0.44		
4,398	N,N-Dimethylaminoethanol	0.00	0.00	34.25	*p*-Cou	[62]
4,687	Butane,1,2:3,4-diepoxy-,	0.60	1.80	0.00		
4,558	Ethanamine, 2-chloro-N,N-dimethyl-	0.00	0.00	6.16		
5,272	Propanenitrile, 2-(dimethylamino)-	0.00	0.00	0.19		
5,653	Furfural	2.86	2.73	0.20	Cell/ Hemi	
5,938	2-Furanmethanol	0.62	0.69	0.41	Cell/ Hemi	
6,085	2-Propanone, 1-(acetyloxy)-	0.36	0.29	0.30	Cell/ Hemi	
6,741	Acetic acid, 2-(dimethylamino)ethyl ester	0.00	1.11	0.78	Hemi	
7,513	Butanedioic acid, cyclic hydrazide	0.87	1.93	0.00		

8,081	N-Butyl-tert-butylamine	3.92	6.96	0.73		
8,622	3(2H)-Furanone, 4-hydroxy-5-methyl-	1.15	2.03	0.00	Cell/ Hemi	
10,268	4H-Pyran-4-one, 2,3-dihydro-3,5-dihydroxy-6-methyl-	0.17	0.00	0.00		
11,302	Benzofuran, 2,3-dihydro-	0.00	1.88	0.00	Cell/ Hemi	
11,938	Saccharide	0.00	11.59	0.00		
11,081	N,N-Dimethylaminoethanol	0.00	0.00	1.57		
12,680	2-Methoxy-4-vinylphenol	4.31	3.67	0.26	lignin	[64]
12,826	Saccharide	0.00	6.58	0.00		
13,492	L-Proline, 5-oxo-, methyl ester	0.00	0.00	0.00	protein	[64]
15,312	ß-D-Glucopyranose, 1,6-anhydro-	5.86	5.33	1.29	Cell	[65]
20,172	n-Hexadecanoic acid	9.76	4.54	0.00	Lignin	[66]
15,662	L-Glutamine	0.00	0.00	0.53	protein	
18,764	Cyclo(L-prolyl-L-valine)	0.00	0.00	0.16	protein	
19,914	Pyrrolo[1,2-a]pyrazine-1,4-dione, hexahydro-3-(2-methylpropyl)-	0.00	0.00	0.61	protein	[66]
20,067	5,10-Diethoxy-2,3,7,8-tetrahydro-1H,6H-dipyrrolo pyrazine	0.00	0.00	0.83		
20,142	n-Hexadecanoic acid	0.00	0.00	0.92	Hemi	[67]
21,781	10E,12Z-Octadecadienoic acid	8.99	0.41	0.24	Lipid	[64]
21,856	cis-Vaccenic acid	5.98	0.64	0.52	Lipid	[64]
22,050	Octadecanoic acid	2.16	0.53	0.74	Lipid	[64]
23,141	Dimethylaminoethyl palmitate	0.00	0.00	0.13		
23,414	2-((8Z,11Z)-Heptadeca-8,11-dien-1-yl)-4,5-dihydrooxazole	0.53	0.00	0.00		
25,022	Hexadecanoic acid, 2-hydroxy-1-(hydroxymethyl)ethyl ester	1.25	0.46	0.00	Lipid	

30,855	ß-Sitosterol	1.27	0.50	0.00	lignin glycoside	[68]

Pyrolysis – 550 °C

RET. TIME (min.)	COMPOUND	RAW Rel. Area (%)	HTE-GLY Rel. Area (%)	HTE-MA Rel. Area (%)	ORIGIN	REFERENCE
1,816	Carbon dioxide	10.72	9.50	15.24		
1,970	Methanethiol	0.00	0.00	4.06		
2,143	Butane	4.02	3.83	4.12		
2,589	Acetic acid	3.48	2.91	0.88	Cell/ Hemi	
2,670	Furan, 3-methyl-	1.08	0.99	0.92	Cell/ Hemi	
3,194	2-Propanone, 1-hydroxy-	1.61	0.96	1.07		
3,684	Furan, 2,5-dimethyl-	0.54	0.44	0.65	Cell/ Hemi	
4,020	Butanenitrile, 3-methyl-	0.00	0.00	0.33	Protein	[69]
4,276	Pyrrole	0.77	0.84	0.65		
4,537	Toluene	3.17	2.39	3.51		
4,718	Succindialdehyde	0.24	0.07	0.22	Lignin	
4,792	Propanoic acid, 2-oxo-, methyl ester	1.04	0.55	0.82		
4,973	Pyrrolidine, 2-butyl-1-methyl-	0.72	0.94	0.89		
5,231	3-Furaldehyde	0.34	0.28	0.33	Cell/ Hemi	
5,493	Furfural	1.55	1.16	0.72	Hemi	
6,377	Styrene	0.23	0.25	0.59		
6,605	2(5H)-Furanone	0.25	0.00	0.50	Cell/ Hemi	
6,803	1,2-Cyclopentanedione	1.22	1.16	1.17	Cell/ Hemi	
7,428	Butanedioic acid, cyclic hydrazide	0.77	1.50	0.00		
7,010	N-Methylmaleimide	0.00	0.00	0.29		

7,626	Phenol	0.53	0.59	0.79	Phenol	
8,000	N-Butyl-tert-butylamine	2.03	2.59	0.00		
8,357	1,2-Cyclopentanedione, 3-methyl-	0.49	0.40	0.59		
8,551	3(2H)-Furanone, 4-hydroxy-5-methyl-	0.22	0.80	0.00	Cell/ Hemi	
9,115	Phenol, 3-methyl-	1.60	1.86	1.89	Lignin	
9,357	Phenol, 2-methoxy-	1.02	0.91	0.53	Lignin	
9,711	Maltol	0.22	0.29	0.31		
10,517	Phenol, 4-ethyl-	0.30	0.38	0.28	Lignin	
10,792	4H-Pyran-4-one, 3,5-dihydroxy-2-methyl-	0.30	0.52	0.40		
10,925	Creosol	1.28	1.24	0.27	Phenol	
11,289	Benzofuran, 2,3-dihydro-	0.86	1.17	1.14	Cell/ Hemi	
12,023	Saccharide	0.00	2.12	0.00		
12,153	Phenol, 4-ethyl-2-methoxy-	0.44	0.23	0.00	Lignin	
12,299	1,2-Benzenediol, 4-methyl-	0.24	0.00	0.00	Lignin	
12,455	Indole	0.56	0.55	0.38		
12,673	2-Methoxy-4-vinylphenol	1.69	1.67	0.45	Lignin	[64]
13,139	2,3-Dimethoxyphenol	0.41	0.39	0.00	Lignin	
13,734	1H-Indole, 7-methyl-	0.40	0.40	0.57		
14,380	3,5-Dimethoxy-4-hydroxytoluene	0.55	0.86	0.00		
14,493	Phenol, 2-methoxy-4-(1-propenyl)-	0.30	0.50	0.00	Guaiacol-type	
15,299	ß-D-Glucopyranose, 1,6-anhydro-	15.13	21.69	23.07	Cell	
15,856	Phenol, 4-ethenyl-2,6-dimethoxy-	0.99	0.37	0.00	Syringyl-type	[65]
16,309	1,6-Anhydro-a-d-galactofuranose	0.46	0.00	0.63	Cell/ Hemi	

17,455	Phenol, 2,6-dimethoxy-4-(2-propenyl)-	0.26	0.25	0.16	Lignin	
17,781	Ethanone, 1-(4-hydroxy-3,5-dimethoxyphenyl)-	0.23	0.22	0.00		
18,142	Syringylacetone	0.06	0.00	0.00	Syringyl-type	
18,200	Pyrrolo[1,2-a]pyrazine-1,4-dione, hexahydro-	0.17	0.32	0.33		
18,754	Cyclo(L-prolyl-L-valine)	0.11	0.00	0.17		
19,914	Pyrrolo[1,2-a]pyrazine-1,4-dione, hexahydro-3-(2-methylpropyl)-	0.30	0.00	0.61	protein	
20,070	5,10-Diethoxy-2,3,7,8-tetrahydro-1H,6H-dipyrrolo pyrazine	0.77	0.32	1.33		
20,145	n-Hexadecanoic acid	2.84	3.91	1.04	Lipid	[64]
21,785	10E,12Z-Octadecadienoic acid	1.56	0.42	0.32	Lipid	[64]
21,839	cis-Vaccenic acid	1.59	1.04	0.84	Lipid	[64]
22,043	Octadecanoic acid	0.62	0.29	0.25	Hemi	
24,043	Pyrrolo[1,2-a]pyrazine-1,4-dione, hexahydro-3-(phenylmethyl)-	0.32	0.27	0.40		
29,288	ß-Sitosterol acetate	0.44	0.25	0.00	lignin glycoside	[68]
30,855	ß-Sitosterol	0.31	0.15	0.00	lignin glycoside	[68]

Supplementary material References

62. Xin, Y.; Shen, X.; Dong, M.; Cheng, X.; Liu, S.; Yang, J.; Wang, Z.; Liu, H.; Han, B. Organic Amine Mediated Cleavage of Caromatic-Cαbonds in Lignin and Its Platform Molecules. Chem. Sci. 2021, 12, 15110–15115, doi:10.1039/d1sc05231d.
63. Carrier, M.; Windt, M.; Ziegler, B.; Appelt, J.; Saake, B.; Meier, D.; Bridgwater, A. Quantitative Insights into the Fast Pyrolysis of Extracted Cellulose, Hemicelluloses, and Lignin. ChemSusChem 2017, 10, 3212–3224, doi:10.1002/cssc.201700984.
64. Sobek, S.; Zeng, K.; Werle, S.; Junga, R.; Sajdak, M. Brewer's Spent Grain Pyrolysis Kinetics and Evolved Gas Analysis for the Sustainable Phenolic Compounds and Fatty Acids Recovery Potential. Renew. Energy 2022, 199, 157–168, doi:10.1016/j.renene.2022.08.114.
65. Somorin, T.; Parker, A.; McAdam, E.; Williams, L.; Tyrrel, S.; Kolios, A.; Jiang, Y. Pyrolysis Characteristics and Kinetics of Human Faeces, Simulant Faeces and Wood Biomass by Thermogravimetry–Gas Chromatography–Mass Spectrometry Methods. Energy Reports 2020, 6, 3230–3239, doi:10.1016/j.egyr.2020.11.164.

66. Dandamudi, K.P.R.; Muhammed Luboowa, K.; Laideson, M.; Murdock, T.; Seger, M.; McGowen, J.; Lammers, P.J.; Deng, S. Hydrothermal Liquefaction of Cyanidioschyzon Merolae and Salicornia Bigelovii Torr.: The Interaction Effect on Product Distribution and Chemistry. Fuel 2020, 277, 118146, doi:10.1016/j.fuel.2020.118146.
67. Machado, L.M.M.; Lütke, S.F.; Perondi, D.; Godinho, M.; Oliveira, M.L.S.; Collazzo, G.C.; Dotto, G.L. Simultaneous Production of Mesoporous Biochar and Palmitic Acid by Pyrolysis of Brewing Industry Wastes. Waste Manag. 2020, 113, 96–104, doi:10.1016/j.wasman.2020.05.038.
68. Ahmad, S.R.; Ghosh, P. A Systematic Investigation on Flavonoids, Catechin, β-Sitosterol and Lignin Glycosides from Saraca Asoca (Ashoka) Having Anti-Cancer & Antioxidant Properties with No Side Effect. J. Indian Chem. Soc. 2022, 99, 100293, doi:10.1016/j.jics.2021.100293.
69. Chen, H.; Shan, R.; Zhao, F.; Gu, J.; Zhang, Y.; Yuan, H.; Chen, Y. A Review on the NOx Precursors Release during Biomass Pyrolysis. Chem. Eng. J. 2023, 451, 138979, doi:10.1016/j.cej.2022.138979.
